# Hippocampal Transcriptome Profile of Persistent Memory Rescue in a Mouse Model of *THRA1* Mutation-Mediated Resistance to Thyroid Hormone

**DOI:** 10.1038/srep18617

**Published:** 2016-01-08

**Authors:** Yiqiao Wang, André Fisahn, Indranil Sinha, Dinh Phong Nguyen, Ulrich Sterzenbach, Francois Lallemend, Saїda Hadjab

**Affiliations:** 1 Department of Neuroscience, Karolinska Institutet, 171 77 Stockholm, Sweden; 2 Neuronal Oscillations Laboratory, Division for Neurogeriatrics, Center for Alzheimer Research, NVS Department, Karolinska Institutet, 14186 Stockholm, Sweden; 3 Department of Biosciences and Nutrition, Karolinska Institutet, 14157 Huddinge, Sweden

## Abstract

Hypothyroidism due to *THRA1* (gene coding for thyroid hormone receptor α1) mutation-mediated Resistance to Thyroid Hormone (RTH) has been recently reported in human and is associated with memory deficits similar to those found in a mouse model for *Thra1* mutation mediated RTH (*Thra1*^+/*m*^ mice). Here, we show that a short-term treatment of *Thra1*^+/*m*^ mice with GABA_A_ receptor antagonist pentylenetetrazol (PTZ) completely and durably rescues their memory performance. In the CA1 region of the hippocampus, improvement of memory is associated with increased in long-term potentiation (LTP) and an augmentation of density of dendritic spines (DDS) onto the apical dendrites of pyramidal cells reflecting an increase in the local excitatory drive. Unbiased gene profiling analysis of hippocampi of treated *Thra1*^+/+^ and *Thra1*^+/*m*^ mice were performed two weeks and three months post treatment and identified co-expression modules that include differentially expressed genes related with and predicting higher memory, LTP and DDS in the hippocampi of PTZ-treated animals. We observed that PTZ treatment changed similar sets of genes in both *Thra1*^+/+^ and *Thra1*^+/*m*^ mice, which are known to be involved in memory consolidation and neurotransmission dynamics and could participate in the persistent effects of PTZ on memory recovery.

Thyroid hormone (TH) plays a central role in nervous system development, maturation and function^1^,^2^. Even modest hypothyroidism during gestation induces alterations in the nervous system, which are associated with cognitive deficits, including decreases in memory performances, in infancy and adult life. The physiological effects of TH are mediated by the activation of the distinct nuclear thyroid hormone receptors α (THRA) and THRB that act as ligand-inducible transcription factors, differing in tissue distribution with the brain expressing predominantly THRA1 (80%, encoded by *THRA1* gene). Studies using knock out strategies for the distinct THRs reveal that unliganded receptor activity is probably the important factor causing the harmful effects of hypothyroidism^1^. The unliganded receptor activity is mainly due to either deficiency in TH or to a mutation on the THR ligand binding site creating a dominant negative receptor. This mutation leads to resistance to thyroid hormone (RTH) syndrome. While RTH due to *THRB* mutation has been known for decades, it is only recently that patients have been found harbouring *THRA1* mutations, which cause a dominant negative effect of the receptor^3^. However, even though RTH due to *THRA1* is expected to have at least similar prevalence than RTH due to *THRB* mutation (~1 in 40,000)^4^, the lack of specific diagnostic test makes it difficult today to clearly assess the actual number. Patients with a *THRA1* mutation present cognitive dysfunction in childhood and adult life^3^, similar to other syndromes with intellectual and developmental disabilities (IDD). While treatment with high doses of TH might seem an adequate therapy to rescue the metabolic disorders of patients with *THRA1* mutation-induced RTH syndrome, it might not be effective or sufficient to rescue cognitive function^5^.

To study potential alternative therapeutic approaches for the cognitive deficiency due to *THRA1* mutation, we used a mouse line with a *Thra1* mutation resulting in a 10-fold lower affinity to TH^6^. The heterozygous mice (*Thra1*^+/*m*^) present a “receptor-mediated hypothyroidism” in the tissues in which mutant Thra1 is expressed by inhibiting wild-type receptor action in a dominant negative manner. Importantly, the *Thra1*^+/*m*^ mice present all features observed in humans with *THRA* mutation-mediated RTH syndrome (growth retardation, impairment of motor coordination, metabolic changes, skeletal abnormalities and cognitive dysfunction while the circulating hormone level is near-normal), and therefore provide an excellent mouse model for investigating therapeutic treatments of neurological disorders observed in patients with unliganded THRA1 activity or the larger patient population with TH deficiency during development.

In the *Thra1*^+/*m*^ mice, hippocampal network activity is altered and is associated with marked impairments in memory performances^7^,^8^. Moreover, previous work using the *Thra1*^+/*m*^ mice or other hypothyroidism mouse models have shown a severe delay in the development of the GABAergic system in the neocortex and hippocampus of these mice^8^,^9^,^10^. The hippocampus is the site of molecular and anatomical changes that contribute to cognitive functions such as learning and memory processes and anxiety behaviour which depend on the activity dynamics of the different neuronal circuits which are part of its network. Important orchestrators of the activity of neuronal networks are GABAergic interneurons^11^. Within the hippocampal circuit, GABAergic interneurons act to synchronize network activity, a process which is thought to control memory formation^12^. Gamma-aminobutyric acid (GABA), released by GABAergic interneurons, act through two main types of GABA receptors: the ionotropic GABA_A_ receptors (ligand-gated chloride ion channels) and the metabotropic GABA_B_ receptors (G-protein coupled receptors). Both GABA receptors have been largely shown to regulate memory processes, a treatment with antagonists of either receptors increasing memory performance in mice. Specifically, treatments with GABA_A_ receptors antagonists have shown to be very efficient in improving memory performance in several mouse models of syndromes that are characterized by intellectual and developmental disabilities (IDD). For instance, treatment with the non-competitive GABA_A_ antagonists picrotoxin or pentylenetetrazol (PTZ) were shown to ameliorate memory functions in mouse models of the Rett and Down syndromes, respectively^13^,^14^, suggesting that GABA_A_ receptor antagonists may offer a possible therapeutic target for other syndromes associated with memory impairments.

Here, we show that low doses of pentylenetetrazol (PTZ) can rescue completely the memory deficits observed in the *Thra1*^+/*m*^ mice. Importantly, the benefits in learning and memory performances of a short-term treatment persist for several months. Focusing on the CA1 and CA3 regions of the hippocampus, we also observed in the PTZ-treated mutant mice an increase of synaptic strength between CA3 and CA1 pyramidal neurons, together with a subtle increase in spine density quantified on apical dendrites of CA1 pyramidal cells, which are both hallmarks of memory performance^15^,^16^,^17^. Moreover, we identified an increase in genetic pathways (such as the CREB, CAMKIIa and calcium signaling) regulated by PTZ in *Thra1*^+/*m*^ and *Thra1*^+/+^ mice hippocampi and that are commonly associated with memory processes. These changes were still observed in hippocampi of *Thra1*^+/*m*^ and *Thra1*^+/+^ mice three months after PTZ treatment. In addition, unbiased gene pathway analysis revealed predictive augmentation of memory performance and of LTP and density of dendritic spine (DDS) following PTZ treatment, confirming our *in vivo* observations.

## Results

### GABA_A_ receptor antagonism rescues learning and memory deficits in *Thra1* ^+/*m*^ mice

#### Learning and memory

**Figure 1:**
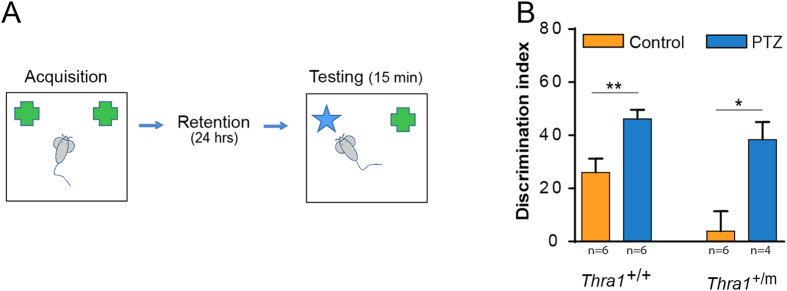
PTZ elicits memory improvement in *Thra1*^+/*m*^ mice. (**A**) Novelty discrimination indices analyzed for *Thra1*^+/+^ and *Thra1*^+/*m*^ mice after 17 days treatment with either PTZ or vehicle. Treatment with PTZ markedly improved the novel recognition task performance in *Thra1*^+/*m*^ (n = 4) and *Thra1*^+/+^ (n = 6) mice compared to control condition (n = 6 *Thra1*^+/*m*^ and n = 6 *Thra1*^+/+^). (**B**) All values are mean ± S.E.M. *P < 0.05, **P < 0.01, Two tailed, unpaired *t*-test.

Several studies have shown that in intellectual and developmental disability (IDD) mouse models with learning and memory impairment similar to those observed in *Thra1*^+/*m*^ mice^8^, the use of an GABA_A_ antagonist can rescue memory performance^14^,^18^,^19^,^20^. In line with this we assessed whether a low (non-epileptic) dose of pentylenetetrazol (PTZ, 5 mg/kg/day, 17 days of treatment), a non-competitive GABA_A_ receptor antagonist, could rescue memory performance in *Thra1*^+/*m*^ mice. The dose of PTZ used in the study did not promote spontaneous seizure development. Moreover, the chronic treatment with PTZ did not changed the mice sensitivity in regard to proconvulsant dose of PTZ (40mg/kg) regardless of the genotype (data not shown). We used the novel object recognition task to measure visual recognition memory^21^. This test represents a model of declarative memory that depends on hippocampal function^14^,^22^ (Fig. 1A). It takes advantage of a rodent’s natural tendency to explore novel objects; an animal showing performant memory would recognize the familiar object and spend more time in exploring the novel object. Importantly, locomotor ability is not a critical factor for this task, and therefore does not influence performance of the animals in this test. To proceed to the acquisition phase of the test, *Thra1*^+/+^ and *Thra1*^+/*m*^ animals were trained for 5 days (15 min/day) during which they could explore two identical objects. There was no significant difference between genotypes in the exploratory preference of the two similar objects, with a discrimination index not different from “0” for both *Thra1*^+/+^and *Thra1*^+/*m*^ animals (*P* > 0.05). For the testing session, one of the familiar objects used in the training session was replaced with a novel object. As indicated in Fig. 1B, *Thra1*^+/+^ animals showed a marked preference in exploring the novel object, while in contrast *Thra1*^+/*m*^ mice showed similar preference for both the novel and the familiar objects. Indeed, the discrimination index in *Thra1*^+/*m*^ mice was still close to “0” whereas it was about 26% in the *Thra1*^+/+^ cohort (DI: control *Thra1*^+/*m*^ 3.8 ± 7.6, n = 6 *versus* control *Thra1*^+/+^ 26 ± 5.2, n = 6, *P* = 0.036), indicating that *Thra1*^+/*m*^ mice do not discriminate between the familiar and the novel objects placed in the arena while *Thra1*^+/+^ mice do. These data confirm previous results demonstrating a memory deficit in this mouse model of TH deficiency^8^. Remarkably, our chronic PTZ treatment (5 mg/kg/day, 17 days of treatment) reversed completely the detrimental effect of the *Thra1* mutation on learning and memory functions (DI: PTZ *Thra1*^+/*m*^, 38.3 ± 6.4, n = 4 *versus* control *Thra1*^+/+^, 26 ± 5.2, n = 6, *P* = 0.17) and improved memory performance in the *Thra1*^+/+^ (DI: control *Thra1*^+/+^, 26 ± 5.2, n = 6 *versus* PTZ *Thra1*^+/+^, 46.2 ± 3.4, n = 6, *P* = 0.008). Indeed, no significant difference in the discrimination index was observed after PTZ treatment when comparing *Thra1*^+/+^ PTZ with *Thra1*^+/*m*^ PTZ groups (Fig. 1B).

We next assessed whether the memory recovery observed in *Thra1*^+/*m*^ mice following a short term treatment with low dose of PTZ was limited only to the period following the treatment. To test this, *Thra1*^+/+^ PTZ and *Thra1*^+/*m*^ PTZ groups were kept untreated for 3 months and evaluated for their memory performance in the novel object recognition task. Strikingly, the complete learning and memory recovery observed in *Thra1*^+/*m*^ mice immediately after PTZ treatment was sustained 3 months later, indeed no significant difference were observed between those 2 groups [Discrimination index (D.I.) in 3 months post treatment PTZ-treated *Thra1*^+/*m*^ mice: 45.9 ± 3.2; n = 4 versus DI in PTZ-treated *Thra1*^+/*m*^ mice: 38.3 ± 3.7; n = 4; *P* = 0.34]. These results indicate that short-term treatment with GABA_A_ antagonists induces persistent improvement of learning and memory performances.

### GABA_A_ receptor antagonism enhances long-term potentiation and dendritic spine density in *Thra1* ^+/*m*^ mice hippocampus

The CA3-CA1 synapses of the hippocampus have been intensively studied in relation to learning and memory processes. These synapses are glutamatergic (excitatory) and constitute a simplified neuronal model to investigate neuronal network activity in response to PTZ treatment.

#### Long-term potentiation

**Figure 2:**
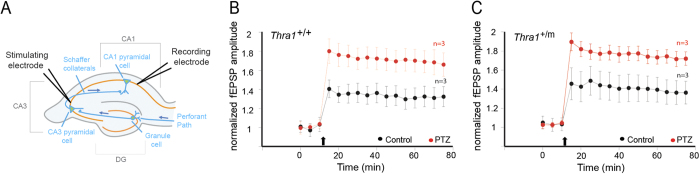
PTZ elicits Long-term Potentiation. (**A**) Schematic representation of hippocampal slice and the positioning of stimulating and recording electrodes. (**B**) PTZ treatment-dependent differential increase of field EPSP amplitude after theta burst-stimulation (black arrows) in *Thra1*^+/+^ and *Thra1*^+/*m*^ hippocampus. Field EPSP amplitude in *Thra1*^+/+^ hippocampal area CA1 from animals treated with vehicle (black; n = 3) or PTZ (red; n = 3) (left panel). Field EPSP amplitude in *Thra1*^+/*m*^ hippocampal area CA1 from animals treated with vehicle (black; n = 3) or PTZ (n = 3; red) (right panel).

LTP is the electrophysiologically recorded increase of synaptic strength between synaptically connected neurons and is considered a hallmark measurement of learning and memory performances^17^. Hence, treatment with drugs that enhance LTP has been correlated positively with learning and memory, whereas drug-induced blocking of LTP has been associated with decreased learning and memory performance^17^. Here, we recorded LTP in CA1 *stratum pyramidale* after theta burst stimulation of CA3 area in the same animals that previously received a treatment with low dose of PTZ or vehicle and performed the novel object recognition task (see Fig. 1). Surprisingly, we found that there was no difference in fEPSP (field Excitatory Post-Synaptic Potentials) amplitude between *Thra1*^+/+^ and mutant groups before treatment (Fig. 2B vehicle, black circles). However, treatment of *Thra1*^+/+^ and *Thra1*^+/*m*^ animals with PTZ for 17 days significantly increased LTP in both groups (Fig. 2, PTZ, red circles). Those results indicate that PTZ treatment increases the synaptic strength between CA3-CA1 pyramidal neurons and that this might *in vivo* participate in the cellular signature of PTZ on learning and memory rescue in the *Thra1*^+/*m*^ mice.

#### Effect of PTZ on dendritic spines density

**Figure 3:**
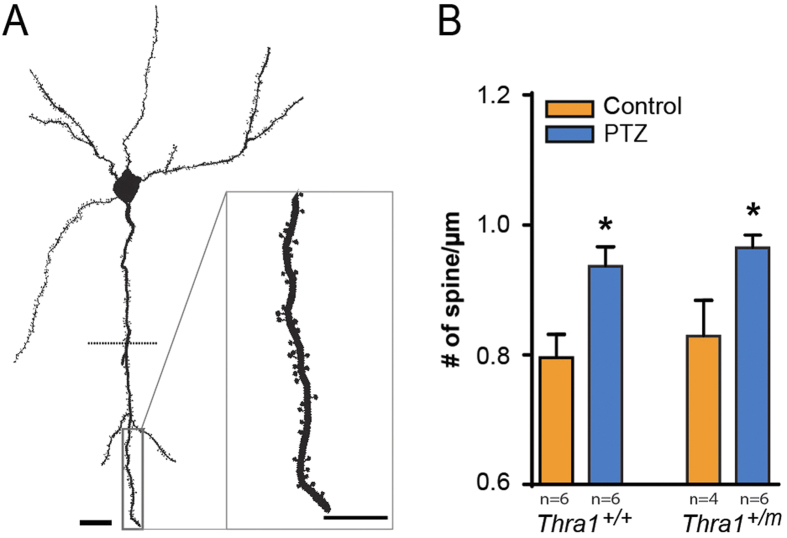
PTZ elicits an increase of density of dendritic spines. (**A**) Neurolucida based drawing of Golgi-cox impregnated CA1 pyramidal cell with spines. Dash line underlines the first bifurcation point of the apical dendrite in the drawing. Inset show a higher magnification of a spiny dendritic segment. (**B**) PTZ treatment increase the number of spine in the apical tuft of the pyramidal cell in *Thra1*^+/+^ (n = 6) and *Thra1*^+/*m*^ (n = 4) mice compared with control groups (*Thra1*^+/+^, n = 6 and *Thra1*^+/*m*^, n = 6). Scale bars (20 μm and 10 μm in inset).

**Figure 4:**
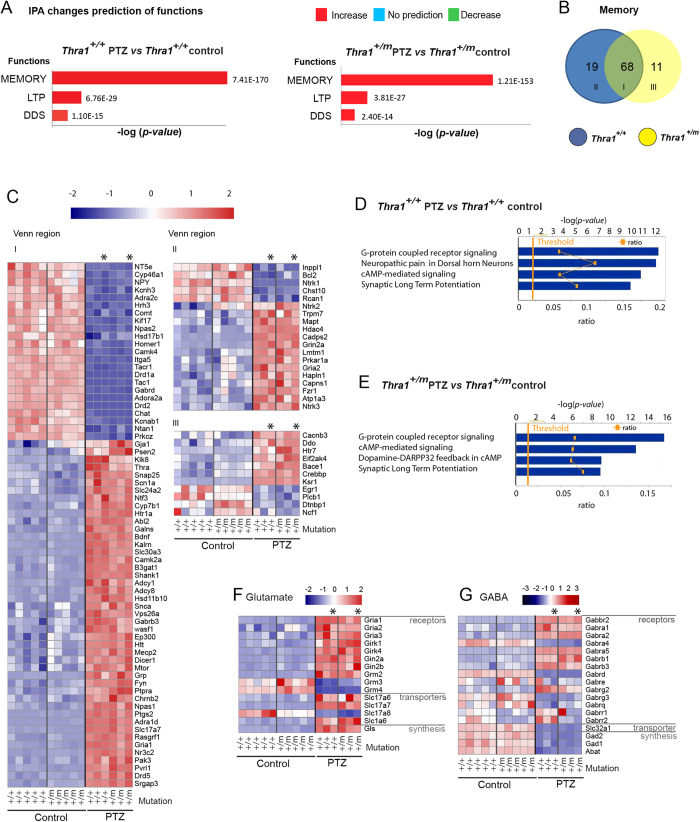
IPA-based genes associated with the improvement of memory performance by PTZ. (**A**) IPA-based gene ontology selected functions with z-score >2 for memory, long-term potentiation (LTP) and density of dendritic spine (DDS) from *Thra1*^+/+^ PTZ vs. *Thra1*^+/+^ control (left panel; n = 3 and n = 5 respectively) and *Thra1*^+/*m*^ PTZ vs. *Thra1*^+/*m*^ control (right panel; n = 3 and n = 5 respectively). (**B**) Venn diagram representing genes overlapping or not for the memory function between *Thra1*^+/+^ PTZ vs. *Thra1*^+/+^ control and *Thra1*^+/*m*^ PTZ vs. *Thra1*^+/*m*^ control (*Thra1*^+/*m*^) mice. (**C**) Heatmap depicts gene expression value of Venn regions I-III: genes up-regulated by PTZ treatment are colored in shades of red; genes down-regulated are colored in shades of blue (*Thra1*^+/+^ control; ^+/+^ n = 5, *Thra1*^+/+^ PTZ, ^+/+^, n = 3; *Thra1*^+/*m*^ control, ^+/m^, n = 5 and *Thra1*^+/*m*^ PTZ, ^+/m^, n = 3 (note that PTZ animals 3 months post-treatment are quoted with *). (**D**,**E**) Predicted canonical pathways trigger by PTZ treatment analyzed in IPA. The ratio represent the number of genes from our data set divided by the total number of genes that map to the same pathway in IPA (**F**,**G**) Heatmaps of genes encoding Glutamatergic (**F**) and GABAergic (**G**) receptors, vesicular transporters and synthesizing enzymes (selected by literature search).

The antagonistic action of PTZ on GABA_A_ receptors is expected to increase excitatory drive in the CA3-CA1 circuit, which is supported by our LTP recordings (Fig. 2). To assess whether this is correlated with a change in the number of synapses, dendritic spine density (DDS, which form upon excitatory synaptic connection on the postsynaptic dendrite) on the apical dendrites of CA1 pyramidal cells were analyzed. Counting of spine density in both *stratum radiatum* and *stratum lacunosum-moleculare* (CA1) between *Thra1*^+/+^ and *Thra1*^+/*m*^ mice showed no difference before treatment (Fig. 3B, *P* = 0.62). However, PTZ treated groups showed significant increase in the number of spines (Fig. 4C) (number of spine/μm: control *Thra1*^+/+^, 0.83 ± 0.055, n = 6 *versus* PTZ *Thra1*^+/+^, 0.94 ± 0.023, n = 4, *P* = 0.023; control *Thra1*^+/*m*^, 0.80 ± 0.036, n = 6 *versus* PTZ *Thra1*^+/*m*^, 0.96 ± 0.019, n = 6, *P* = 0.04). Those results suggest that the low dose PTZ treatment might enhance the formation of new dendritic spines and that this increase in spines number could participate in the improved learning and memory performances after PTZ treatment in *Thra1*^+/+^ and *Thra1*^+/*m*^mice. Moreover, analysis of spine shapes showed that PTZ treatment significantly increased small and large spine number. Indeed, PTZ treatment increased the number of thin and stubby spines (thin spines/μm: control, 0.065 ± 0.024, n = 5 *versus* PTZ, 0.15 ± 0.023, n = 4, *P* = 0.0025 and stubby spines/μm: control, 0.24 ± 0.05, n = 5 *versus* PTZ, 0.37 ± 0.034, n = 4, *P* = 0.007) but not the mushroom spines (mushroom spines/μm: control, 0.32 ± 0.067, n = 5 *versus* PTZ, 0.38 ± 0.038, n = 4, *P* = 0.22).

### Persistent changes in hippocampal gene expression profile following low dose PTZ treatment

**Figure 5:**
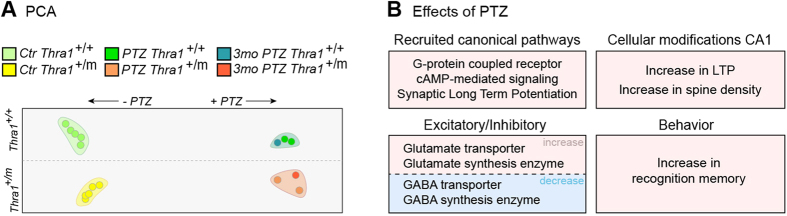
Schematic illustration of the mechanism of action of PTZ at the hippocampal level in *Thra1*^+/+^ and *Thra1*^+/*m*^ mice. (**A**) Principal Components Analysis (PCA) using R of the memory category. Clouds were arbitrary made to underline the shift of the plots with the PTZ treatment [short-term and long-term, 3 months (3mo)] in *Thra1*^+/+^ and *Thra1*^+/*m*^ mice (detail of gene changes in Fig. 4 and Suppl. data). Note that in the memory category, PTZ treatment does not rescue gene expression in *Thra1*^+/*m*^ *versus Thra1*^+/+^ mice but modifies gene expression similarly and sustainably in both genotypes. (**B**) In the left panels, effects of PTZ on the recruitment of canonical pathways and on the expression of genes that control excitability in the hippocampus. Data derive from gene expression performed in the hippocampus of both *Thra1*^+/+^ and *Thra1*^+/*m*^ mice. On the right panels, effects of PTZ on cellular modifications at CA1 and on recognition memory behavior in mice treated with PTZ. Light pink and light blue in panels represent increase and decrease in the observed changes, respectively.

To investigate the short- and long-term effects of our PTZ treatment on the transcriptional profile of hippocampus from *Thra1*^+/*m*^ and *Thra1*^+/+^ mice, we performed full-transcriptome sequencing of hippocampi from *Thra1*^+/*m*^ and *Thra1*^+/+^ mice 2 weeks and 3 months post-treatment (Fig. 5). Focusing on the treatment effect and using a q-value of 0.01 for analysis we observed that following the treatment 3082 genes and 2774 genes in *Thra1*^+/+^ and *Thra1*^+/*m*^ groups, respectively, were significantly (*P* < 0.05) either up-or down-regulated.

The biological functions that are expected to be increased or decreased according to the gene expression changes in our dataset were identified using the IPA regulation z-score algorithm (detailed in Materials and Methods section). With our data set filtered out for “Functions for Nervous system Development and Function”, functions such as memory, LTP and DDS were predicted to be significantly increased by PTZ treatment in both groups by expressing a z-score >2 (Fig. 4A, for detailed gene list see Supplementary Dataset), corroborating our *in vivo* data obtained for memory (Fig. 1), LTP (Fig. 2B) and DDS (Fig. 3B). In the memory function category (Fig. 4B), PTZ treatment changed the expression of 87 genes in *Thra1*^+/+^ and 79 in the mutants, and 34 and 31 of those, respectively in *Thra1*^+/+^ and mutant groups, showed a modification of expression that was consistent with a net increase in memory performances (Fig. 4A). Interestingly, comparing the *Thra1*^+/*m*^ with *Thra1*^+/+^ groups, we could also find that the treatment affected mostly the same set of genes (Fig. 4B), and in the same direction regardless of the genotype (Fig. 4C). Importantly, those genetic modifications were still observed 3 months after the end of the treatment (Fig. 4C,F,G, the asterisk).

Next, focusing on the memory function category, we investigated the canonical pathways predicted to be involved. Notably, similar canonical pathways in *Thra1*^+/+^ groups and *Thra1*^+/*m*^ groups were found to be triggered by the PTZ treatment: G-coupled receptor signaling, c-AMP-mediated signaling and LTP signaling (Fig. 4D) as well as CREB signaling in neurons, Ca^2+^ signaling and Neurotrophin/Trk signaling (a detailed gene list is provided in Supplementary Dataset).

To investigate the function of GABA_A_ antagonism on pathways involved in the neurotransmission dynamics, we analyzed genes coding for the GABA and GLU pathways, which represent the main inhibitory and excitatory neurotransmitters, respectively, in the brain. We focused on synthesis enzymes, vesicular transporters and receptors. We observed that the synthesis enzymes for GABA (*Gad1* and *Gad2*) were down-regulated by the treatment while *Gls*, the GLU synthesizing enzyme, was up-regulated (Fig. 4F,G). Similarly, the vesicular transporters for GABA (*Slc32a1*) and for glutamate (*Slc17a6*, *Slc17a7* and *Slc1a6*) were down- and upregulated, respectively. At the receptor level, after PTZ treatment, results for GABA receptors were more divided; 5 subunits of GABA receptors were downregulated while 7 others were upregulated in *Thra1*^+/*m*^ group (Fig. 4G). For Glutamate, most AMPA and Kainate receptors (*Gria1*, *Gria2*, *Gria3*, *Grik1*, *Grik4*, *Gin2a*, *Gin2b* and *Grm2*) were up-regulated (Fig. 4F). Taken together, these results demonstrate that PTZ treatment dramatically and persistently changes gene expression patterns in the hippocampus and that all observed changes could contribute in rescuing durably the memory performance of *Thra1*^+/*m*^ mice (Fig. 5).

## Discussion

Congenital or developmental hypothyroidism lead to delayed postnatal development along with reduced memory performance^1^. Memory impairments are usually associated with alterations in the hippocampal inhibitory circuit that is under control of the GABAergic system. Here we provide evidence that a short-term treatment with low (non-epileptogenic) doses of a GABA_A_ receptor antagonist (PTZ) completely restores learning and memory performance in a mouse model of TH deficiency mediated by *Thra1* mutation. Strikingly, this complete rescue of memory behaviour remained normalized 3 months after treatment. Results obtained in hippocampal slices of *Thra1*^+/+^ and *Thra1*^+/*m*^ mice showed that antagonism of GABA_A_ receptors increased LTP, an excitatory response driver required for memory^23^ as well as excitatory spine density in the CA1 hippocampal region, which is known to correlate with increased memory performance. Moreover, full-transcriptome data from hippocampus of *Thra1*^+/+^ and *Thra1*^+/*m*^ mice treated with low dose of PTZ identified genes and canonical pathways presumably involved in the improvement of memory function affected by PTZ. Strikingly, these genes changes were also still noticed 3 months post-treatment. Thus, this study identifies short term treatment with low doses of GABA_A_ antagonists as potential therapeutic agents for alleviating intellectual disabilities in TRα-mediated RTH or developmentally hypothyroid patients.

GABA_A_ receptors are ligand-gated ion channels complexes that bind the more potent inhibitory neurotransmitter in the central nervous system, GABA, resulting in chloride influx and consequent reduction of neuronal excitability. The specificity of connections and actions of this inhibitory system has been suggested to directly participate in the synchronization of neuronal activity that is one of the underlying mechanisms that participate in memory formation^12^. In this context, many studies have focused on adjusting the activity of the GABAergic system with the objective to affect memory performance. Hence, a decrease of GABA-mediated inhibition using GABA_A_ receptor antagonists has been associated with enhanced memory consolidation while its increase using GABA_A_ receptor agonists, with impaired memory function^24^,^25^,^26^. In our experiments, the rescue of memory impairments observed in our hypothyroid mouse model is obtained using chronic administration of the GABA_A_ receptor antagonist PTZ for a defined period of time. Such treatment has been hypothesized to decrease GABA-mediated inhibition and consequently to increase the excitatory/inhibitory (E/I) ratio throughout the brain, including the hippocampus^14^. This ratio has been suggested to contribute to the network homeostasis that is crucial for proper brain function and for which the disruption might lead to syndromes characterized by IDD^27^. A decrease in the E/I ratio has been proposed indeed for many syndromes associated with a loss of memory performance^14^, including the DS, and for which similar treatment that the one used in our study has been demonstrated to ameliorate memory deficits^13^,^14^. Remarkably, the positive effects of the chronic administration of PTZ on learning and memory functions in the *Thra1*^+/*m*^ mice extend long beyond the time of treatment. Similar long lasting rescue of memory functions has been observed with PTZ in a DS mouse model^14^. This indicates that if a treatment with low doses of PTZ could improve memory performance via a reestablishment of the network homeostasis through a decrease of the inhibitory drive, which could account for a short term improvement, the persistence of these positive effects implies long lasting changes in the neuronal circuits that cannot be attributable to simply repressing inhibitory synapses over the time of treatment. Rather, specific molecular mechanisms must have been activated which resulted in permanent (or durable at least) changes. Remarkably, our data indicate that such molecular changes, assessed by gene expression profiling in the hippocampus, not only occur right after PTZ treatment, but remains three months later, irrespective of the genotype of the animal analysed. More specifically, our data show that the enzymes synthesizing GABA and its vesicular transporter are down-regulated at the end of the PTZ treatment while it is the opposite for glutamate. These changes in gene expression could conceivably participate in correcting the network homeostasis. Moreover, throughout the hippocampus, glutamate and GABA receptor subunits are reorganized. Hence, even though further investigations are required at the single cell level to identify the cell type specific modifications, our data suggest that our PTZ treatment could induce a cellular switch in neurotransmitter and neurotransmitter receptor identity which, ultimately, could also participate in regulating the network dynamics of the hippocampus and eventually memory functions.

It is generally assumed that memory involves cellular changes. LTP is widely accepted as a cellular basis of the hippocampus-dependent memory^23^,^28^,^29^,^30^. In our study, we show that LTP at the CA1 region increases after PTZ treatment, which is in accordance with previous studies and suggests a cellular basis for the observed learning/memory improvements. However, before treatment our data show that CA1 LTP was not significantly different between *Thra1*^+/+^ and *Thra1*^+/*m*^ mice. This lack of changes in LTP at the CA1 region in normal conditions has been reported in other mouse models of IDD such as for Fragile X syndrome (FXS) or DS^14^,^31^,^32^,^33^,^34^. On the other hand, in these mouse models, LTP recorded from the same animals at the perforant path-GC was impaired in mutants^14^,^35^,^36^. These results suggest that for some IDD, the LTP in CA1 may not be significantly changed compared to a control group despite clear learning memory impairment in the IDD mouse models. In contrast, a reduction of LTP in the dendate gyrus is commonly reported in IDD mouse models. Such changes could occur in the *Thra1*^+/*m*^ mice and would require further investigation. Nevertheless, it is worth noting that LTP in CA1 and dentate gyrus has been suggested to have distinct physiological relevance. LTP in DG would be involved in filtering and transferring information (requiring a strong signal) and the maintenance of short-term memory^37^. In contrast, LTP in the CA1 region, which is induced more easily, may contribute to the formation of more solid and long-lasting memory^37^. It is therefore conceivable that LTP facilitation observed in the CA1 region after decreased efficiency of GABA-mediated inhibition in PTZ-treated animals forms part of the cellular basis responsible for the long-lasting memory improvements observed in treated *Thra1*^+/*m*^ mice.

Cellular structural plasticity changes such as dendritic spine density have been shown to be related to memory^15^,^16^. In theory, enhanced spine formation would promote increased learning and memory performance. In the mature hippocampus, dendritic spines form the primary sites of excitatory synaptic transmission, and their number and morphology play an important role in synaptic plasticity^38^. LTP has been shown to induce dendritic spine structural modifications^39^,^40^, including new spine formation^41^. CA1 LTP is induced by coordination of presynaptic (Schaffer collaterals, which are CA3 pyramidal neuron axons) and postsynaptic (dendritic arborization of hippocampal CA1 pyramidal neurons) activity. On the apical side, CA1 pyramidal neurons receive synaptic inputs from Schaffer collaterals proximal to the soma and from the entorhinal cortex (via the perforant path) on distal dendrites. We report here an increase in density of dendritic spines in CA1 pyramidal neuron’s apical tree that is presumably related to the increase of LTP due to the antagonistic action of PTZ on GABA_A_ receptors, which is expected to increase excitatory drive in the neuronal network and/or correlate to new memory formation.

Potential molecular signatures for memory in the hippocampus have been under investigation for a long time. Many genes have been identified and are involved in synaptic transmission, neuronal architecture and cell signalling molecules. In our hippocampus data set, apart from the genes coding for neurotransmitters or their receptors and discussed previously, we could identify key canonical pathways triggered by PTZ treatment and leading to up-regulation of major components of memory. Brain-derived neurotrophic factor (BDNF), G-protein coupled receptor signalling, Ca^2+^ and cAMP-mediated signalling and CREB signalling in neurons as well as Neurotrophin/Trk signalling are all canonical pathways relevant to memory function^42^,^43^ and that were found to be upregulated in our data set. Several first messengers and their G-protein coupled receptors or (Ca^2+^) ligand/voltage dependent (Na^+^/Ca^2+^) ion channels (NMDA *grin2a*, which have their activity facilitated by AMPA receptor *gria1* and *gria2*) are known to increase c-AMP and Ca^2+^. More specifically at the second messenger production level, Ca^2+^-stimulated type 1 and type 8 adenylate cyclases (*adcy1* and *adcy8*), main potent producers of cAMP, are also up-regulated. Ca^2+^/calmodulin-dependent protein kinase 2a (*camk2a*) but not CAMK4 (*camk4*) is up-regulated. Moreover, cAMP response element–binding protein, CREB co-activator P300 (*ep300*) and CREB binding protein CBP (*crebbp*) were also up-regulated. The main target of CREB complex related to memory is brain-derived neurotrophic factor (*Bdnf*), which is a potent modulator of synaptic transmission and plasticity in the CNS, acting both pre- and postsynaptically, and is up-regulated together with its receptor TrkB (*Ntrk2*) in our data set after PTZ treatment. Many genes involved in these pathways and found to be upregulated after PTZ treatment (including *camk2*, *bdnf*, *ntrk2*, *crebbp*, *ep300*, *adcy1*, *adcy8*, *grin2a*, *gria1* and *gria2*) have been shown to be decisive components in memory formation, synaptic neurotransmission, long-term potentiation and/or spine formation^44^,^45^,^46^,^47^,^48^,^49^,^50^,^51^,^52^,^53^,^54^,^55^,^56^,^57^. Particularly, our study suggests that PTZ treatment might recruit the CaMKIIα-BDNF-CREB–mediated synaptic plasticity pathway to improve memory in a long lasting manner. Interestingly, these pathways are upregulated in the hippocampus after PTZ treatment regardless of the mouse genotype, in both *Thra1*^+/+^ and *Thra1*^+/*m*^ mice. This suggests that PTZ does not rescue the hippocampal genetic profile of the mutant mice but does rescue their memory phenotype by recruiting genes whose function is to ameliorate memory performance, bypassing thus the original behavioral failure.

PTZ has a broad spectrum of actions that conceivably may act synergistically to provide the positive effect on memory by establishing a new long-lasting homeostasis in the hippocampal circuits. Patients harbouring the mutation in the *THRA* gene are just beginning to be found but the incidence of the mutation is expected to be at least similar to that of the RTH due to *THRB* mutation^4^. A new parameter of identification has been recently established: elevated T3/T4 ratio and T3/rT3, which should aid in the identification of more patients in the near future. Moreover, thyroid hormone treatment has been inefficient in counteracting the cognitive deficits observed in patients presenting *THRA1* mutation-induced RTH^5^. Therefore, both the need and the potential for effective pharmacotherapy to alleviate memory deficits in patients with *THRA1* mutation-mediated RTH are real. Currently clinical trials are in progress for GABA_A_ specific antagonist R04938581 or highly selective GABA_A_α5 negative allosteric modulator to ameliorate learning/memory performances in patients with DS.

The benefit of PTZ on memory and neuronal plasticity together with its potential neuroprotective effect via BDNF may also argue in favor of considering GABA_A_ antagonist therapy for neurological disorders associating memory deficit to neurodegeneration such as Alzheimer’s disease. Further identification of gene expression changes of neuronal populations using single cell RNAseq from specific hippocampal areas and tracing experiments will enable us to better understand the specific site and mode of action of GABA_A_ antagonists in regard to memory.

## Material and Methods

### Animals

The *Thra1*^+/*m*^ and their control littermates *Thra1*^+/+^ mice were used in the present study. The *Thra1*^+/*m*^ mouse strain carrying the dominant negative R384C mutation in the *Thra1* gene has been described previously^6^. Animal care procedures were in accordance with the guidelines set by the European Community Council Directives (86/609/EEC). All experimental protocols and animals required were approved by the local ethical committee “Stockholms norra djurförsöksetiska nämnd”. In the current study adult male mice (3 to 9 months old) were used. All efforts were made to minimize the number of mice used.

### Treatment

#### Low dose PTZ

*Thra1*^+/+^ and *Thra1*^+/*m*^ mice were administered Pentylenetetrazol (PTZ), a GABA_A_ receptor antagonist via daily oral feeding (5.0 mg/kg in milk) for 17 days. This represents a non-epileptic dose that can be safely given to rodents for up to 1 year^58^. In total, *Thra1*^+/+^ and *Thra1*^+/*m*^ mice received 17 daily doses of milk (referred to as Control) or a milk-PTZ (referred to as PTZ).

### Novel object recognition task

The novel object recognition task is based on the innate tendency of rodents to differentially explore novel objects over familiar ones. Experiments were conducted in the morning. Mice had been individually submitted to daily handling sessions, and been given an opportunity to habituate to a black open-field arena (35 × 35 × 15 cm, cage size). In order to minimize the anxiety-related behavior that the *Thra1*^+/*m*^ mice exhibit in a novel environment, each mouse had been trained and tested in its own arena containing some bedding from their home cage. After the 5 days of habituation to the arena, mice were exposed to two identical objects (porcelain figurines) positioned in two corners of the apparatus (5 cm from the wall) during a 15 min training session for 5 consecutive days. We used 15 min instead of the more commonly used 5 min because of the high anxiety and low locomotion exerted by the mutant mice. Testing sessions were conducted 24 h after the training session and mice were exposed to the object they had explored the previous days as well as to a new object (metallic ball, 4 cm diameter).

Operationally memory was defined as the proportion of time animals spent investigating the novel object minus the proportion of time spent investigating the familiar one. It was calculated as follows: Discrimination Index, DI = [(Novel Object Exploration Time/Total Exploration Time)–(Familiar Object Exploration Time/Total Exploration Time)] × 100. Exploration included any investigative behavior (i.e. head orientation, sniffing occurring within <2.0 cm) or deliberate contact that occurred with each object. The objects are cleaned with 90% ethanol, dried, and ventilated for a few minutes between mice to avoid any olfactory influence. Exploratory behavior was assessed by an experimenter via video transmission on a screen at 3 meters from the experiment site to not disturb the animal behavior. Data are reported as Mean ± Standard Error of the Mean (S.E.M). Two‐tailed, Student’s unpaired *t*‐test was used for statistics.

### Electrophysiology

Electrophysiological experiments were performed in hippocampal slices of *Thra1*^+/+^ and *Thra1*^+/*m*^ mice (3.5 months old) from PTZ and control groups. Mice were decapitated and the brain rapidly removed and placed in ice-cold artificial cerebrospinal fluid (ACSF, in mM: KCl 2.49, NaH_2_PO_4_ 1.43, NaHCO_3_ 26, glucose 10, sucrose 252, CaCl_2_ 1, MgCl_2_ 4). The brain was then hemisected, trimmed and horizontal 350 μm-thick hippocampal slices were obtained using a vibratome (Leica V1000). Slices were kept in a submerged holding chamber containing ACSF (in mM: NaCl 124, KCl 3.5, NaH_2_PO_4_ 1.25, MgCl_2_ 1.5, CaCl_2_ 1.5, NaHCO_3_ 30, glucose 10), constantly bubbled with 95% O_2_ and 5% CO_2_, incubated at 35 °C for one hour and then allowed to cool to room temperature. Local Field Potential recordings were carried out in *stratum pyramidale* of hippocampal subfield CA1 at 28 °C in an interface recording chamber using borosilicate microelectrodes containing ACSF (resistance 3–5 MΩ) and a MA102 amplifier (Cologne University, Germany). A concentric bipolar stimulation electrode was placed in *stratum pyramidale* of hippocampal subfield CA3. Stimulation intensity for each slice was chosen as the lowest level of stimulation voltage that evoked a discernible population spike. Electrical stimulation was given as follows: Baseline – 5 times every 1 min for 10 min; Theta Burst-Stimulations (TBS) – 3 burst trains with 20 s interval with each train consisting of 10 × 5 Hz-series of 4 × 100 Hz pulses of 400 μs duration; first 10 min after TBS–5 times every 1 min for 10 min; next hour–1 time per min for 60 min. LTP analysis was carried out in ClampFit (Molecular Devices, USA) and figures constructed in KaleidaGraph (Synergy Software, Reading, PA, USA) using values for the amplitude of the field EPSP. Data is reported as Mean ± SEM. Student’s unpaired t-test was used for statistics.

### Golgi-cox

Whole brains of *Thra1*^+/*m*^ and *Thra1*^+/+^ mice from PTZ or control groups were removed and Golgi-cox impregnation and staining were performed according to the manufacturer’s protocol (FD Rapid Golgi Staining, FD Neurotechnologies, Ellicott City, MD, USA). Coronal sections of 250 μm thickness were cut on a cryostat, mounted onto slides, cleared with ethanol and xylene and cover-slipped. Three-dimensional reconstructions of neurons were performed blind to genotype and treatment using 100X oil immersion objective with bright field on a Zeiss microscope/stage: Axio Imager M1 equipped with a computer-controlled motorized stage and Neurolucida (version 10).

For analysis, dendritic segments were selected from apical dendrites of CA1 pyramidal cells from brain sections that were from similar coronal planes at a random distance from the first branch point, in the apical tuft (after primary apical dendrite bifurcates). We excluded superficially positioned neurons to ensure that dendritic trees were intact. A total number of 269 segments (n = 4–6 animals in each group), 25–30 μm–long with a diameter between 0.4–1.0 μm were analysed in blind of treatment and genotype condition. The number of spines and the thickness of the dendrites were analyzed using Neurolucida explorer (version 10). Note that no correction factor has been applied. Spines were classified by their shape; we counted mushroom-shaped spines with well-formed head and neck structures, stubby spines, thin spines and filopodia-like structures as defined by Neurolucida software.

### TruSeq RNA sequencing and data analysis

Hippocampi from PTZ-treated (PTZ) or untreated (control) *Thra1*^+/+^ or *Thra1*^+/*m*^ (5 *Thra1*^+/+^ control, 5 *Thra1*^+/*m*^ control, 3 *Thra1*^+/+^ PTZ-treated and 3 *Thra1*^+*/m*^ PTZ-treated) were dissected at 2 weeks and 3 months post treatment, and following behavior analysis. RNA was extracted using a Qiagen mini kit. Succinctly, to convert RNA into a sequenceable library, mRNA have been purified and fragmented, cDNA have been produced, adapter ligated, purified and quantified following the low-throughput protocol from TruSeq RNA sample preparation guide. Single-end RNA-seq reads of 51 nucleotides in length were generated on an Illumina HiSeq 2000 sequencer according to the manufacture’s protocol. The sequence reads were mapped to the mouse NCBI37/mm9 genome build and normalized data was generated for each genomic feature using STRT software^59^. In STRT software raw reads were aligned using Bowtie. RPKM (reads per KB per million reads) normalization method^60^ was used to normalize the mapped reads whereas unmapped reads were removed. For differential expression, normalized signal values <3 in at least one type of mouse RNA-sample and differential expression p-value >0.05 were excluded from the analysis. After filtering out genes average expression level was calculated and 1.2 fold up and down regulated gene lists were generated for different criteria as follows: 1/*Thra1*^+*/*+^ PTZ *vs Thra1*^+*/*+^ control, 2/*Thra1*^+*/m*^ PTZ *vs Thra1*^+*/m*^ control and 3/*Thra1*^+*/m*^ control *vs Thra1*^+*/*+^ control.

To identify biological functions affected by either the mutation or the treatment, we used Ingenuity Pathway Analysis (IPA, Ingenuity^®^ Systems). The RPKM normalized filtered data were tabulated and uploaded into the IPA web application (www.ingenuity.com). To increase stringency in order to identify the top biological functions associated with either the mutation or the treatment, we used a false discovery rate (q-value) equal to 0.01 (over 100 genes, 99 are true positive). The biological functions that are expected to be increased or decreased according to the gene expression changes in our dataset were identified using the IPA regulation z-score algorithm which reveals the statistical measure of correlation between gene expression and relationship direction. A positive or negative z-score value indicates that a function is predicted to be increased or decreased respectively. Moreover, a function with a z-score ≥2 or ≤−2 is considered as a function which is significantly increased or decreased respectively. From the selected lists we have filtered out few functional categories such as memory, LTP, DDS and listed the genes involved in each category. To view the molecular relationship between molecules and identify canonical pathways present in each selected functional category, we used IPA’s connect tool and interaction information based on ingenuity Knowledge Database for each list. We used Qlucore Omics Explorer (www.qlucore.com) for initial data analysis and “pheatmap” package from R Bioconductor (http://www.bioconductor.org) to perform a hierarchical clustering analysis and generated heatmaps for memory related category of genes and for glutamate and GABAergic signaling.

## Additional Information

**How to cite this article**: Wang, Y. *et al.* Hippocampal Transcriptome Profile of Persistent Memory Rescue in a Mouse Model of *THRA1* Mutation-Mediated Resistance to Thyroid Hormone. *Sci. Rep.* **6**, 18617; doi: 10.1038/srep18617 (2016).

## Supplementary Material

Supplementary Dataset
